# Eosinophilic esophagitis prevalence, incidence, and presenting features: a 22-year population-based observational study from southwest Sweden

**DOI:** 10.1093/dote/doae025

**Published:** 2024-03-24

**Authors:** J Plate, T Söderbergh, J Bergqvist, C Lingblom, H Bergquist, H Larsson

**Affiliations:** Department of Otorhinolaryngology Head and Neck Surgery, Region Västra Götaland, NU-Hospital Group, Trollhättan, Sweden; Department of Otorhinolaryngology, Head and Neck Surgery, Institute of Clinical Sciences, Sahlgrenska Academy, University of Gothenburg, Gothenburg, Sweden; Department of Otorhinolaryngology Head and Neck Surgery, Region Västra Götaland, NU-Hospital Group, Trollhättan, Sweden; Pulmonary Department, Sleep Disorders Centre, Sahlgrenska University Hospital, Gothenburg, Sweden; Centre for Sleep and Wake Disorders, Sahlgrenska Academy, Gothenburg University, Gothenburg, Sweden; Department of Infectious Diseases, Institute of Biomedicine, The Sahlgrenska Academy, University of Gothenburg, Gothenburg, Sweden; Department of Clinical Microbiology, Sahlgrenska University Hospital, Gothenburg, Sweden; Department of Otorhinolaryngology, Head and Neck Surgery, Institute of Clinical Sciences, Sahlgrenska Academy, University of Gothenburg, Gothenburg, Sweden; Department of Otorhinolaryngology Head and Neck Surgery, Region Västra Götaland, NU-Hospital Group, Trollhättan, Sweden; Department of Otorhinolaryngology, Head and Neck Surgery, Institute of Clinical Sciences, Sahlgrenska Academy, University of Gothenburg, Gothenburg, Sweden

**Keywords:** eosinophilic esophagitis, incidence, prevalence, swallowing disorders

## Abstract

Eosinophilic esophagitis (EoE) is a chronic inflammatory condition of the esophagus that affects both children and adults. Symptoms in adults are mainly esophageal dysphagia, which ranges from mild symptoms to acute food bolus obstruction of the esophagus. Diagnosis is defined as symptoms of esophageal dysfunction and ≥ 15 eosinophils/high power field (HPF) in at least one of the biopsies taken from the esophagus. EoE appears to be increasing in both prevalence and incidence. The aim of this study was to investigate the prevalence, incidence, and presenting symptoms of patients with EoE within the catchment area of Northern Älvsborg County Hospital in Trollhättan. Patient records with the ICD code of EoE between 2012 and 2022 and pathology reports from esophageal biopsies from 2000–2022 were examined. Patients with symptoms of esophageal dysfunction and > 15 eosinophils/HPF were classified as having EoE. In total, 409 EoE patients (379 adults and 30 children) fulfilled the diagnostic criteria during the follow-up period. The overall prevalence was 113 cases/100 000 inhabitants (adults 127/100 000 and children 57/100 000) at 31 December 2022. The incidence was 7/100 000 and increased during the observation period. At diagnosis, 46% of the adults and 11% of the children had a history of acute bolus obstruction requiring hospitalization, while 51% of adults and 22% of children exhibited endoscopic findings of fibrosis. The prevalence of EoE is significantly higher than that generally reported in an area of southwest Sweden. The results indicate that the incidence is increasing; however, whether this is due to an actual increase or heightened awareness of EoE is inconclusive. Acute bolus obstruction is a common presenting symptom among EoE patients and is most likely an effect of late diagnosis.

## Graphical Abstract

**Graphical Abstract:**
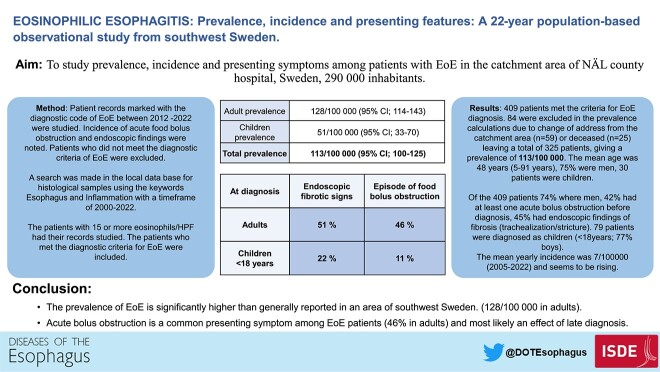

## INTRODUCTION

Eosinophilic esophagitis (EoE) is a relatively new disease of the esophagus. The entity of EoE was first described in the middle of the 1990s.^1^^,^^2^ EoE is a chronic type 2 inflammatory condition that is driven by food and/or aeroallergens. There is evidence indicating that EoE is part of the atopic march,^3^ and patients with EoE often have other allergic comorbidities, such as allergic rhinitis and/or asthma.

Symptoms in adults are mainly esophageal dysphagia but can also present as acute food bolus obstruction of the esophagus or, more rarely, other signs of esophageal dysfunction, such as regurgitation, vomiting, or pain while swallowing. In younger children, abdominal pain, vomiting, and feeding dysfunction are more commonly presenting features.^4^

EoE is the most common cause of esophageal dysphagia, especially among otherwise young and healthy people, and it is the second most common cause of inflammation in the esophagus next to gastroesophageal reflux disease (GERD). ^(^^5^^)^

The diagnosis of EoE requires both symptoms of esophageal dysfunction and esophageal biopsies with ≥15 eosinophils/high power field (HPF).^6^ Endoscopists usually identify characteristic signs of fibrosis (such as trachealization and strictures) and/or inflammation (including furrows, white plaque, and edema) during gastroscopy. Intriguingly, endoscopic findings do not always correlate with the level of inflammation.^6^ There is a risk that biopsies are not collected during the examination if the physician is unaware of the lack of correlation between endoscopic findings and the level of inflammation. Diagnostic delays frequently occur over several years due to both patient- and doctor-related reasons. EoE patients develop coping mechanisms to compensate for their difficulties in swallowing, such as slower eating, chewing carefully, and excessive water intake during meals, while general practitioners do not promptly refer patients to endoscopy or that biopsies are not taken during endoscopy by the endoscopist.^7^ Several studies have shown that symptoms of EoE negatively affect patients’ quality of life, and EoE patients describe a feeling of high emotional burden, especially because of the long duration between the onset of symptoms and diagnosis.^8–10^

The reported prevalence of EoE in the USA and Europe is approximately 30–55 patients per 100 000 inhabitants, although a Spanish study from 2018 reported a prevalence as high as 112/100 000.^11–17^

The aim of this study was to assess the prevalence, incidence, and presenting features in children and adult EoE patients in an area of southwest Sweden with a population of approximately 290 000.

## MATERIALS AND METHODS

### Study setting

This study was performed in the southwest part of Sweden defined as the catchment area of Northern Älvsborg County Hospital (NÄL), NU-hospital group, which is a part of the greater region of Västra Götaland. The area is predominantly rural, interspersed with a few larger towns with a population of up to 60 000 inhabitants. As of 31 December 2022, the total population in this region was 288 410 inhabitants (data were obtained from ‘Statistiska centralbyrån’ (SCB, Statistics sweden). The home address of the patients was monitored to assure that they were still living in the catchment area.

There is a possibility for the inhabitants to apply for care within the greater region that includes Gothenburg, Skövde, and Lidköping hospitals. However, this needs active management from the patients and is unusual.

There are no other caregivers performing diagnostic procedures in terms of gastroscopy in the investigated area; therefore, all performed gastroscopies can be assumed to have been performed at NÄL hospital. In addition, biopsies taken from the gastroscopic procedure are routinely sent to the pathology department located at the same hospital.

### Patient selection

The diagnosis of EoE was based on the presence of $\ge$15 eosinophils/HPF in at least one of the biopsies collected from the esophagus as well as concurrent symptoms of esophageal dysfunction (mainly dysphagia) noted in the medical records.

Patient records marked with the “International Statistical Classification of Diseases and Related Health Problems” code (ICD: K209. A) (a specific diagnostic code for the disease that was introduced in 2012), EoE between 2012 and 2022 was studied regarding both symptoms and pathology reports. Patients who did not meet these diagnostic criteria for EoE were excluded.

In addition, a search in the local pathology database was performed using the keywords: Location: Esophagus (T62) and diagnostic code: Inflammation (M4) with a time frame of 2000–2022. The patients with an esophageal biopsy with ≥15 eosinophils/HPF had their medical records assessed. The records with information about symptoms of esophageal dysfunction (mainly dysphagia) were included in the study.

### Data extraction

Data regarding sex, time of diagnosis and/or endoscopy, age at diagnosis, mortality status, demography, previous incidence of acute food bolus obstruction in need of hospitalization, comorbidities (GERD, celiac disease, inflammatory bowel disease [IBD], esophageal cancer, allergy), and esophageal endoscopic characteristics (signs of fibrosis: trachealization and strictures) were collected from the medical records.

### Ethics

The study was approved by the National Ethical Review Board (Etikprövningsmyndigheten) DNR-2021-03084 Date: 08 October 2021. The study protocol conforms to the ethical guidelines of the 1975 Declaration of Helsinki as reflected in a priori approval by the institution's human research committee.

### Statistical analysis

Descriptive statistics are presented as the mean value or as a percentage of the total number.

The prevalence of EoE was calculated as the sum of all diagnosed patients still alive and still living in the catchment area on the 31st of December 2022 and then divided by the total population in the catchment area on the same date. The 95% CI was calculated with the standard error of a proportion (*P* ± 1.96 SE(p)) and controlled by a biostatistician using R with exact binominal test function: binom.test.

The annual incidence of EoE was calculated as the number of newly diagnosed cases each year and then divided by the total population in the catchment area for the corresponding year. The results are presented in annual incidence rates per 100 000 inhabitants.

The correlation of incidence numbers was calculated using Pearson’s correlation and presented as r values. *P* values were calculated with a ‘two-tailed test.’

Subgroup analyses were performed for children (<18 years of age) and adults.

All data analysis was controlled by a biostatistician.

## RESULTS

### Study population

In this study, 303 patients were identified by ICD code, of whom 258 met the criteria for diagnosis in the electronic medical record system, 45 lacked eosinophilic inflammation in biopsies or did not have information about esophageal symptoms and were excluded. The pathology record system generated 2314 patients with inflammation in the esophagus, identifying 151 more patients with EoE who were not identified via ICD code. A total of 409 patients fulfilled the criteria for EoE diagnosis in the catchment area between 2001 and 2022, of whom 84 were excluded from the prevalence calculations due to a change in address from the catchment area (*n* = 59) or deceased (*n* = 25), leaving 325 EoE patients at 31 December 2022. The population in the area on 31 December 2022, was 288 410, of which 58 317 were children (<18 years of age) (Sweden statistics).

### Characteristics of the study population

**Fig. 1:**
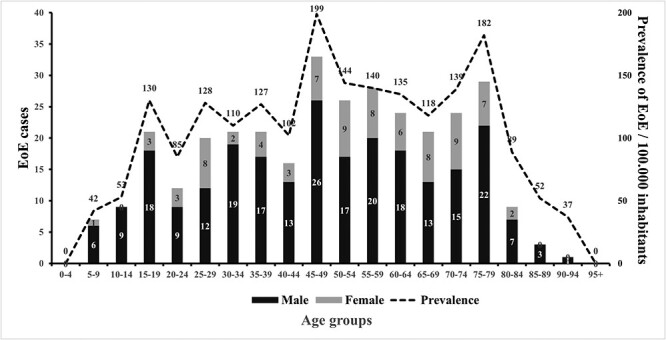
Prevalence by age group and sex in patients diagnosed with eosinophil esophagitis in the catchment area of the NU hospital group, Sweden, on December 31, 2022.

*In the patients constituting the prevalence* (*n* = 325), the mean age was 48 years (range: 5–91 years), 75% (*n* = 245) were men, and 9% (*n* = 30) were children (<18 years of age) (Fig. 1).

**Fig. 2:**
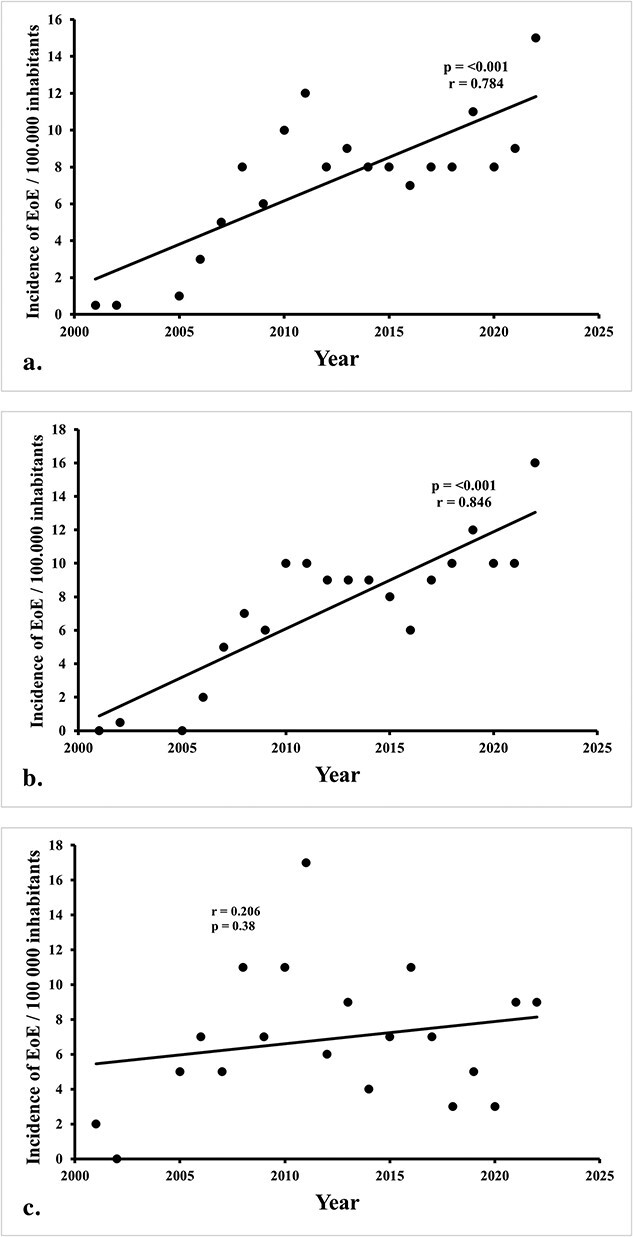
Incidence rates of eosinophil esophagitis during a 22-year follow-up period in southwest Sweden. The figure illustrates the incidence rates in the total population (a), among adults (b), and among children (c).

*In the patients constituting the incidence* (*n* = 409), 74% (*n* = 302) were men, 42% (*n* = 160) had at least one acute bolus obstruction before diagnosis, and 45% (*n* = 184) had endoscopic findings of fibrosis (Fig. 2a, Supplementary Table 1).

Eighty-one percent (*n* = 330) were diagnosed as adults, 73% (*n* = 241) were men, 46% (*n* = 151) had at least one acute bolus obstruction before diagnosis, and 51% (*n* = 167) had endoscopic findings of fibrosis (Fig. 2b, Supplementary Table 1).

Nineteen percent (*n* = 79) were diagnosed as children (<18 years), 77% (*n* = 61) were boys, 11% (*n* = 9) had at least one acute bolus obstruction before diagnosis, and 22% (*n* = 17) had endoscopic findings of fibrosis (Fig. 2c, Supplementary Table 1).

Comorbidities were noted, and 7 patients **(**1.7%) had a concomitant diagnosis of IBD, of which 2 were children, 116 (28%) had GERD, of which 19 were children, 10 (2.5%) had celiac disease, of which 8 were children, 235 (57%) had allergies (seasonal allergy, food allergy and/or eczema), of which 52 were children and 109 (27%) had asthma, of which 29 were children (Table 1).

**Table 1:** Clinical and demographic characteristics of all patients diagnosed with EoE in the catchment area of the NU-hospital group between 2000 and 2022

	Adults ≥18 year (*n* = 330)	Children <18 year (*n* = 79)	Total (*n* = 409)
Age at diagnosis (years)^†^	49 (18–88)	11 (1–17)	42 (1–88)
Male (%)	241 (73)	61 (77)	302 (74)
Previous bolus obstruction (%)	151 (46)	9 (11)	160 (39)
Allergy (%)	183 (55)	52 (66)	235 (57)
Asthma (%)	80 (24)	29 (37)	109 (27)
Endoscopic signs of fibrosis (%)	167 (51)	17 (22)	184 (45)
Trachealization (%)	131 (40)	17 (22)	148 (36)
Strictures (%)	79 (24)	3 (4)	82 (20)
Comorbidities			
IBD (%)	5 (1.5)	2 (2.5)	7 (1.7)
GERD (%)	97 (29)	19 (24)	116 (28)
Celiaki (%)	2 (0.5)	8 (10)	10 (2.5)

^†^Mean **(**min–max)

### Prevalence and incidence of EoE in Southeast Sweden.

The prevalence in the study population on December 31st 2022 was 113/100 000 (95% CI; 100–125). In the adult group, the prevalence was 128/100 000 (95% CI; 114–143) and among children 51/100 000 (95% CI; 33–70).

The total annual mean incidence rate was 7/100 000 (2000–2022) and was increasing (r = 0.784 *P*$\le$0.001) (Fig. 2a). In adults, it was 7.4/100 000 and increasing (r = 0.846, *P*$\le$0.001) (Fig. 2b), in Children, it was 6.9/100 000 and not statistically increasing (r = 0.206, *P* = 0.38) (Fig. 2c).

Dividing the incidence into 6-year periods from when continuous data were available (2005), for the period 2005–2010, the incidence was increasing (r = 0.93, *P* = 0.007), for 2011–2016, the incidence seems to be decreasing (r = −0.79, *P* = 0.06), and for the last period between 2017 and 2022, the incidence seems to again be increasing (r = 0.67, *P* = 0.14).

## DISCUSSION

EoE is still a relatively new disease and for a long time there has been a large lack of knowledge among both physicians and the public, which is most likely the main reason why the duration of time between symptom onset and the established diagnosis for EoE patients is very long. Increased knowledge could be one of the reasons why the incidence and prevalence are increasing, although that is most likely not the only explanation.

In this study, we show that the prevalence of EoE is significantly higher than generally reported^13^ in an area of southwest Sweden of 290 000 inhabitants; this is in line with a study from central Spain in which the authors found a prevalence of 112/100 000.^15^ The common nominator for the two areas is that they both contain active clinicians who also perform research within the field of EoE.

We believe that there is a rather high awareness of EoE among physicians in our catchment area. We have implemented lectures regarding possible endoscopic findings in patients with EoE for physicians who perform endoscopic procedures and introduced a practice in which all patients who seek hospital care due to food bolus obstruction are offered gastroscopies where biopsies are collected and examined to rule out or confirm an EoE diagnosis.

The first diagnosed patient in this study was found as early as 2001. At that time, the first patients did not have a diagnosis of EoE in their medical records; it was only stated that the patients suffered from dysphagia and had eosinophilic inflammation of the esophagus without any further explanation. In 2006, the first patients were diagnosed with EoE as stated in the medical records of our hospital.

As for endoscopic findings, we only included the term ‘stricture’ and have described this as fibrotic signs. Medical records from the early days when the disease first was described are many times difficult to interpret. For example, the mucosa is sometimes described as cobblestone-like or described with the presence of many strictures in a row. The more recent medical records are easier to interpret since the now standardized EREFS score have been used.^18^

The vast majority of the adult patients in this study were diagnosed and treated by physicians at the ear, nose and throat department because it is a standard procedure at the NÄL hospital in which patients with dysphagia and food bolus obstruction are treated by otorhinolaryngologists. It is important that EoE patients are cared for by physicians who have knowledge of EoE. In this study, almost half of the diagnosed patients had been treated at the hospital due to esophageal food bolus obstruction before or at diagnosis, and we speculate that even more had episodes at home that resolved without medical help. This information is in line with EoE patient narratives, where patients reported that they had often complained of esophageal dysphagia to health care providers long before anyone suspects the diagnosis.^19^

In contrast with the study by Lucendo et al.,^15^ the incidence and prevalence in children with EoE were lower in our study. Children are less likely to have total esophageal food bolus obstruction and have more indistinct symptoms^20^, which might explain the unexpectedly low numbers of EoE children in this study. In our hospital, all children are taken care of by pediatric gastroenterologists, and there might be less awareness of the disease as an explanation.

We found an increasing incidence from 2010. The incidence rates in this study are steadily increasing except from approximately 2010–2012, when we see a dramatic increase around the time when the disease received a specific diagnostic code in the electronic medical records.

Allin et al. presented a nationwide analysis regarding the prevalence of EoE in Denmark and found a prevalence of 69.7/100 000 in 2018. A limitation of that study was that their data were collected from a histology register without any data on concurrent symptoms.^16^

A recent Swedish histopathological study found 1412 diagnosed patients nationwide between 2004 and 2015, with an incidence increasing from 1.2 to 2.79 over the last 3 years.^17^ Our numbers are significantly higher.

The comparatively low numbers in the two articles above suggest that the disease also seems to be patchy regarding prevalence and incidence. The patchy prevalence perhaps relates more to higher frequency of EoE-interested physicians rather than environmental factors since the Nordic countries have similar climate, demography, and allergen exposure. In this study the risk of referral bias is low, the endoscopy clinic at NÄL is the only one in the area and we did not include patients living outside our catchment area in the study. Parasitic diseases and hypereosinophilic syndrome are rare in the area and we consider the risk of diagnostic bias as low.

A meta-analysis from 2023 showed a pooled prevalence of 32.7/100 000 (95% CI 31.9–33.4) and incidence numbers of 4.11 per 100 000 (95% CI 4.03–4.19). Also noting the much higher incidence and prevalence rates in the Spanish region consistent with the above-mentioned theory.^11^

In a study from northern Sweden in 2007, 1000 healthy subjects underwent gastroscopy, biopsies were taken from the esophageal mucosa, and 48 of them showed infiltration of eosinophils. Data regarding symptoms of esophageal dysfunction were not presented, but perhaps this is closer to the actual prevalence of EoE.^21^

A strength of this study is the careful control of the data, which was possible due to the readily available information on home address, population and histophatology register; this makes the risk of someone being incorrectly included low.

A limitation of this study is that it is a retrospective study, and we think that there is a large group of undiagnosed EoE patients in our area. We believe there is a need to spread more information about the disease, both to health care professionals and to the public.

## Supplementary Material

Supplement_doae025

## Notes

Financial support: VG-FOUFBD: 977679. Regional funding for local projects.

Specific author contributions: Data curation, Formal analysis, Validation, Writing—original draft, Writing—review & editing: John Plate; Data curation, Validation, Writing—review & editing: T. Söderbergh; J. Bergqvist: Validation, Visualization, Software Analysis, Supervision, Writing—review & editing; C. Lingblom: Methodology, Analysis, Writing, Supervision—review & editing; Henrik Bergquist: Resources, Supervision, Visualization, Writing—review & editing; Helen M Larsson: Conceptualization, Project Management, Methodology, Supervision, Validation, Resources, Writing—original draft, Writing—review & editing.
