# Supplementary material for: Eosinophilic esophagitis prevalence, incidence, and presenting features: a 22-year population-based observational study from southwest Sweden
Source: Dis Esophagus. 2024 Mar 24;38(1):doae025. doi: 10.1093/dote/doae025 (PMC11734667; doi:10.1093/dote/doae025)
Supplement: Supplement_doae025 [file supplement_doae025.docx]

**Supplement**

Supplementary Table 1. Annual incidence of eosinophil esophagitis in children and adults living in the catchment area of the NU hospital group.

| Year | Total  population | Adult  population | Children  (< 18y)  population* | Total population diagnosed | Adults diagnosed | Children  (<18y) diagnosed | Total incidence | Incidence of Adults | Incidence of Children (< 18 y) |
| --- | --- | --- | --- | --- | --- | --- | --- | --- | --- |
| 2001 | 270321 | 210269 | 60052 | 1 | 0 | 1 | 2 | 0 | 2 |
| 2002 | 271033 | 211120 | 59 913 | 1 | 1 | 0 | 0 | 0,5 | 0 |
|  |  |  |  |  |  |  |  |  |  |
| 2005 | 270970 | 212 890 | 58080 | 3 | 0 | 3 | 5 | 0 | 5 |
| 2006 | 271461 | 214 289 | 57172 | 9 | 5 | 4 | 7 | 2 | 7 |
| 2007 | 272045 | 215 718 | 56327 | 14 | 11 | 3 | 5 | 5 | 5 |
| 2008 | 272190 | 216 814 | 55376 | 21 | 15 | 6 | 11 | 7 | 11 |
| 2009 | 272135 | 217 750 | 54385 | 16 | 12 | 4 | 7 | 6 | 7 |
| 2010 | 272315 | 218 699 | 53616 | 28 | 22 | 6 | 11 | 10 | 11 |
| 2011 | 272503 | 219 524 | 52979 | 32 | 23 | 9 | 17 | 10 | 17 |
| 2012 | 273007 | 220 246 | 52761 | 21 | 19 | 3 | 6 | 9 | 6 |
| 2013 | 275048 | 221 663 | 53385 | 26 | 21 | 5 | 9 | 9 | 9 |
| 2014 | 277018 | 222 969 | 54049 | 21 | 19 | 2 | 4 | 9 | 4 |
| 2015 | 279515 | 224 292 | 55223 | 23 | 19 | 4 | 7 | 8 | 7 |
| 2016 | 283842 | 226 756 | 57086 | 19 | 13 | 6 | 11 | 6 | 11 |
| 2017 | 285667 | 227 659 | 58008 | 24 | 20 | 4 | 7 | 9 | 7 |
| 2018 | 287152 | 228 503 | 58649 | 24 | 22 | 2 | 3 | 10 | 3 |
| 2019 | 287987 | 229 181 | 58806 | 31 | 28 | 3 | 5 | 12 | 5 |
| 2020 | 288224 | 229 414 | 58810 | 24 | 22 | 2 | 3 | 10 | 3 |
| 2021 | 288 314 | 229 768 | 58546 | 27 | 22 | 5 | 9 | 10 | 9 |
| 2022 | 288 410 | 230 093 | 58317 | 42 | 37 | 5 | 15 | 16 | 9 |
